# Antibiotic Resistance Patterns of *Listeria* Species Isolated from Broiler Abattoirs in Lusaka, Zambia

**DOI:** 10.3390/antibiotics11050591

**Published:** 2022-04-28

**Authors:** Prudence Mpundu, John Bwalya Muma, Andrew Nalishuwa Mukubesa, Henson Kainga, Steward Mudenda, Flavien Nsoni Bumbangi, Walter Muleya, Patrick Katemangwe, Musso Munyeme

**Affiliations:** 1 Department of Environmental and Occupational Health, Levy Mwanawasa Medical University, Lusaka 33991, Zambia; prudencezimba@gmail.com; 2 Department of Disease Control, School of Veterinary Medicine, University of Zambia, Lusaka 10101, Zambia; jmuma@unza.zm (J.B.M.); mukubesaandrew@gmail.com (A.N.M.); hkainga@bunda.luanar.mw (H.K.); freshsteward@gmail.com (S.M.); bnflavien@gmail.com (F.N.B.); jejifpa@yahoo.com (P.K.); 3 Africa Center of Excellence for Infectious Diseases of Humans and Animals, University of Zambia, Lusaka 10101, Zambia; 4 Department of Veterinary Epidemiology and Public Health, Faculty of Veterinary Medicine, Lilongwe University of Agriculture and Natural Resources, Lilongwe 207203, Malawi; 5 Department of Pharmacy, School of Health Sciences, University of Zambia, Lusaka 50110, Zambia; 6 School of Medicine, Eden University, Lusaka 32379, Zambia; 7 Department of Biomedical Sciences, School of Veterinary Medicine, University of Zambia, Lusaka 10101, Zambia; muleyawalter@gmail.com

**Keywords:** poultry, Lusaka, *Listeria* spp., antibiotic resistance, abattoirs

## Abstract

*L. monocytogenes* is a public health threat linked to fast foods such as broiler chickens. This study aimed to verify the occurrence of *Listeria* species in chickens from abattoirs and evaluate their antimicrobial resistance. In total, 150 broiler carcass swabs distributed as cloacal (*n* = 60), exterior surface (*n* = 60), and environmental (*n* = 30) were collected. *Listeria* species were characterized using biochemical tests and PCR. We conducted antibiotic resistance tests using the disc diffusion and Etest (Biomerieux, Durham, NC, USA) methods. Overall isolation of *Listeria* species was 15% (23/150) 95% CI (10.16–22.33), 2% (3/150) 95% CI (0.52–6.19) and 13% (20/150) 95% CI (8.53–20.08) came from environmental swabs and carcass swabs, respectively. Proportions of positive *Listeria* isolates were *L. monocytogenes* 74% (17/23), *L. welshimeri* 22% (5/23), and *L. innocua* 4% (1/23). *Listeria* species from the exterior carcass swabs was 61% (14/23), cloacal swabs 26% (6/23), and environmental swabs 3% (3/23). *L. monocytogenes* had the greatest resistance percentage to the following antibiotics: clindamycin (61%, 10/23), tetracycline 30% (7/23), and erythromycin 13%, (3/23). Isolation of *L. monocytogenes* in relatively high numbers, including the antimicrobial profiles, suggests a potential risk of the pathogen remaining viable in the food continuum and a public health risk to would-be consumers.

## 1. Introduction

*Listeria* is a facultative anaerobic Gram-positive bacterium with more than 17 recognized *Listeria* species (spp.) [1]. *Listeria* has a high distribution in nature, with previous isolation recorded from humans and several animals in the soil, aquatic plants, and assorted types of foods [2]. *L. monocytogenes* and *L. ivanovii* are known pathogenic strains affecting humans and animals [3]. From the public health point of view, *L. monocytogenes* is the most important *Listeria* spp. that causes a broad spectrum of clinical syndromes, commonly called Listeriosis, with a case fatality of 20–30%. The clinical features range from mild influenza-like illness to meningitis, frequently accompanied by septicemia and meningoencephalitis [3]. *Listeria* is a ubiquitous pathogen, and the common spp. isolated in food environments is *L. innocua* [4]. *L. monocytogenes* are naturally perpetrated and harbored in the environment and food environments where hygienic practices are compromised. It is also known to colonize a variety of foods, mostly of animal origin and less likely in vegetable foods [5]. Most strains of *Listeria* spp. are consumed through meat products, including water [6]. *Listeria* spp. can also be found in foods through cross-contamination, especially in processing plants where prerequisite programs such as Good Manufacturing Practices (G.M.P.s) are not employed [7]. *Listeria* is a fastidious bacterium that can survive in refrigeration facilities and is resistant to commonly used disinfectants found in food premises [8].

Listeriosis is rare compared to other foodborne infections despite high case fatality. It is relatively under-reported due to a lack of surveillance, especially in low-income continents [9]. Accordingly, some studies have shown a low prevalence of 0.05% (1/21) [10], while a prevalence of 63% has been observed by others [11]. While, in other recent studies Schafer and others reported *L. monocytogenes* in breast and thigh samples of poultry as 8.6–44.2%, respectively [12]. *Listeria* spp. was also detected in various poultry products by several other authors [13,14,15,16,17]. In South Africa, *L. monocytogenes* were isolated in raw poultry and meat products at 19.2% and 14.2% [18,19], while, in Zambia, *L. monocytogenes* from vegetables had a 20% prevalence [20].

Identifying *L. monocytogenes* involves culture methods based on selective enrichment and plating. Characterization of specific *Listeria* spp. involves observing colony morphology, sugar fermentation, and hemolytic properties [21]. Although these methods are the gold standard, their major disadvantage is that they are time consuming and may not suit foods with short shelf lives. As a result, more rapid molecular tests such as Polymerase Chain Reaction (PCR) target specific genes such as the highly conserved housekeeping, *prs* gene encoding phosphoribosyl pyrophosphate synthase [22]. These methods have recently been developed [1,23,24], are accurate, have equal sensitivity, rapidly allow testing of the isolates [25,26], and to a greater extent, can reflect the genetic relationship between isolates [27].

Although the *prs* gene does not differentiate different types of spp. of *Listeria*, it nevertheless acts as an indicator of *Listeria* spp. in each sample of interest [22,28].

Infections due to *L. monocytogenes* are becoming increasingly problematic because most of them generally show low susceptibility and are resistant to numerous antibiotics used in both humans and animals. *Listeria* spp., especially the *L. monocytogenes*, are naturally resistant to cephalosporins, and in recent years, antibiotic resistance is becoming one of the biggest problems globally [29]. Broiler chickens are among the foods of animal origin that have recorded a high use of antibiotics because they enhance bird health, production efficiency, and cost [30]. Furthermore, the high antibiotic use in poultry entails increased production demand propelled by consumption. The commonly used antibiotics are but not limited to penicillin G, ampicillin, gentamicin, erythromycin, vancomycin, chloramphenicol, ciprofloxacin, and tetracycline [31]. The Clinical and Laboratory Standards Institute (CLSI) defines the first-line antibiotics used in treating Listeriosis as penicillin G, ampicillin, Trimethoprim/sulfamethoxazole, and imipenem [32]. The use of the same drugs in the treatment of poultry is often practiced [33]. The disproportionate use of antibiotics in veterinary medicine has contributed significantly to the distribution of antibiotic-resistant pathogens in the environment [34] and the detection of antibiotic-resistant *Listeria* spp. in animals, foods, and their environment [35]. Resistant pathogens isolated from food and the environment transmits to humans, and proper measures to prevent their environmental spread are needed.

In the past decade, poultry production has tripled globally [36], and in Africa, the number of slaughtered live birds has increased equally [37]. Most importantly, chickens are a source of protein for many people worldwide, and in the United States, chicken meat consumption exceeds beef or pork [38]. On the other hand, in sub-Saharan Africa, South Africa accounts for 13 times the average per capita poultry consumption and almost three times the average per capita world poultry consumption [37]. Zambia, in recent years, has recorded annual growth rates of about 8% [39], greatly influencing increased poultry consumption due to its perceived economic, safety, and ease of digestion compared to other meat products [40]. Poultry meat sold in smaller pieces that are cheaper encourages consumers to purchase according to what they can afford. In Zambia, Lusaka has increased the demand for chickens, leading to the opening of several chicken abattoirs [41]. With the increase in chicken abattoirs, bacterial contaminants such as *Listeria* and, more importantly, *L. monocytogenes* in the production process have become apparent and, thus, a severe public health risk. Despite the high consumption of poultry and the purported risks, there are limited studies on *Listeria* spp. and their phenotypic antimicrobial resistance, especially in dressed broiler chickens that are among the top meat products readily consumed in the country. This study, therefore, aimed to determine the prevalence of *Listeria* species isolated from poultry abattoirs located within Lusaka and to determine the resistant patterns of the isolated *Listeria* spp.

## 2. Results

### 2.1. Prevalence of Listeria spp.

Out of 150 samples, 30 from environmental swabs and 120 from poultry carcasses, the prevalence of *Listeria* spp. was 15% (23/150) (Table 1).

### 2.2. Description and BLAST Analysis of Listeria spp.

To confirm the type of *Listeria* spp. isolated, BLAST analysis was performed based on the similarity scores ranging from 97% to 100% with reference sequences. Out of the 23 *Listeria* spp. sequences, 17, 1, and 5 identified as *L. monocytogenes*, *L. innocua*, and *L. welshimeri*, respectively. (Table 2).

### 2.3. Specific Listeria spp. Isolation across Different Abattoirs

When specific abattoir establishment contamination using the total positive samples is considered, abattoir A had the highest contamination of *L. monocytogenes* with 65% (15/23), while abattoir B reported a higher prevalence of *L. welshimeri* at 13% (3/23). Meanwhile, abattoir B is the only one that recorded contamination of *L. innocua* in environmental swabs at a prevalence of 4% (1/23). There was no *Listeria* spp. isolated from abattoir C (Table 3).

### 2.4. Isolation of Listeria spp. According to Surface Swabbed

Concerning surface swabbed, 61% (14/23), 26% (6/23), and 13% (3/23) of the *Listeria* spp. isolated were exterior, cloacal, and environmental swabs, respectively. (Table 4).

### 2.5. Distribution of Antimicrobial Resistance Listeria spp. among Abattoirs

Among the antimicrobial resistant isolates of *Listeria* spp. from abattoirs A and B, the most common resistance phenotype was clindamycin (*n* = 14/23). The distribution is as follows: *L. monocytogenes* (*n* = 10/23) isolated from abattoir A, *L. innocua* (*n* = 1/23) isolated from abattoir B, and *L. welshimeri* (*n* = 3/23) isolated from abattoir B. Resistance to tetracycline was also recorded in abattoir A (*n* = 7/23) of which all isolates were *L. monocytogenes*. In addition, erythromycin resistance was also recorded (*n* = 5/23) and distributed as follows: *L. monocytogenes* (*n* = 3/23) isolated from abattoir A and *L. welshimeri* (*n* = 2/23) isolated from abattoir B. While vancomycin showed susceptibility on both the disc and Etest methods, chloramphenicol, gentamicin, and levofloxacin only showed susceptibility on the Etest method (Table 5).

## 3. Discussion

This study isolated *Listeria* spp. from poultry abattoirs located within Lusaka Province and determined A.M.R. *Listeria* spp. in poultry carcasses. Further, this study found an overall prevalence of 15% (23/150) of *Listeria* spp. While, the antimicrobials that recorded the highest resistance in *Listeria* spp. were clindamycin 61% (14/23), tetracycline 30% (7/23), and erythromycin 22% (5/23). The most significant finding of this study is the isolation of *L. monocytogenes* as the significant contaminant in poultry dressed carcasses, with a prevalence of 74% (17/23). To the best of our knowledge, this study is the first in Zambia to isolate and investigate the antibiotic-resistant patterns of *Listeria* species isolated from dressed poultry in abattoirs within Lusaka Province. *Listeria* spp. especially *L. monocytogenes*, which is responsible for Listeriosis infection, is an important foodborne pathogen [3], and it is known to colonize a variety of food products, including poultry. Several studies have shown that chicken meat is one of the foods involved as a vehicle for *L. monocytogenes* [42,43,44] infection, ranging from 15.8%, 20%, and 58%, respectively [45,46,47]. Previous reports recorded lower levels of isolation; while the results of this current study were on the higher side than previously reported, isolation variations was due to differences in slaughter processes since the sampled chickens in this study did not come from the same abattoir. Introduction of *L. monocytogenes* into meat products is possible during processing and, depending on the hygienic measures by food handlers, cross-contamination occurs at this stage [47,48]. Additionally, most chickens sampled in this study were kept under refrigeration temperatures, allowing *L. monocytogenes* to multiply [48]. In this current study, abattoirs that isolated several *Listeria* spp. were characterized by improper cleaning of the environment and utensils such as knives. *Listeria* spp. can form biofilms at refrigeration temperature; thus contributing to the persistence and dissemination of the pathogen [47].

*Listeria* spp. isolated from two poultry abattoirs using traditional culture and biochemical tests were identified as: *L. monocytogenes*, *L. innocua,* and *L. welshimeri* by sequencing and BLAST analysis of the *prs* gene Table 2. Although the *prs* gene does not differentiate the different *Listeria* spp. on PCR, based on the similarity scores obtained from BLAST analysis results, we infer with some level of confidence, the most likely spp. of *Listeria* that could have been present in the samples Table 2. This combined approach thus ensured that the isolates under study identified up to spp. level, *L. monocytogenes*, and *L. innocua* have been among the common *Listeria* spp. in foods and processing environments [49,50]. However, the results of this study indicated that *L. welshimeri* was the second-highest contaminant (22%) (Table 3). Other authors found different results from this study, with *L. monocytogenes* and *L. innocua* being the significant contaminants in most sampled foods and processing plants [42]. These differences could be linked to variances in the sampled foods from the two studies and differences in environmental factors, isolation method, and sample size.

In this current study, *L. monocytogenes* had the highest prevalence of contamination, 74%, compared to other isolated species of *Listeria*. It is a severe public health concern, especially if the strains belong to serotypes mainly implicated in Listeriosis and if they are allowed to contaminate other raw foodstuffs due to poor or lack of food safety management systems [28]. Commonly, most Listeriosis cases are reported from high-income countries, while cases are much more likely to go unreported in countries where surveillance systems are lacking. *Listeria* cases mainly occur sporadically and are reported more in high-income countries, where incidence is relatively low but has a high fatality rate [51]. Several outbreaks of ready-to-eat foods (R.T.E.) have come from processing plants in the past years. For instance, Effimia et al. (2015) reported a 14.4% prevalence of *L. monocytogenes* in R.T.E. foods products in Greece, while Wu and co-workers observed a 20% prevalence in retail shops in China [51,52]. The world’s biggest Listeriosis outbreak happened in South Africa, and it originated from processing plants of R.T.E. foods [53].

Interestingly, in this current study, *L. monocytogenes* and *L. innocua* appeared clustered according to specific abattoirs, implying some endemic existence in these specific abattoirs. Equally, different contamination levels were recorded in different abattoirs in this study, with one abattoir recording no *Listeria* contamination. *Listeria* spp. contamination is common in processing plants that have compromised hygienic standards, more importantly, if prerequisite programs such as G.M.P.s and Standard Operating Systems (S.O.P.s) are lacking [54]. *Listeria* spp. are post-processing contaminants indicative of lapses in the entire production system [55]. Evidence shows that poultry carcasses may become contaminated either environmentally during production or from healthy birds in the processing abattoirs [56]. Equally, abattoir designs and equipment used in processing plants significantly influence the quality of food products [57]. A study on poultry abattoirs also found contamination levels that differed from one abattoir [58]. They mainly attributed the differences in the prevalence of contamination to be structurally related to any other factor. These highlighted differences could partly explain why some abattoirs had higher contamination levels than others. Mainly abattoir C did not record any *Listeria* contamination because they had a functioning food control system against *Listeria* contamination. As earlier mentioned, *Listeria* spp. are post-processing contaminants that thrive in environments with poor hygienic processes.

This study revealed lower *Listeria* spp. contaminations in the environmental swabs compared to carcass swabs. The finding is attributable to the poultry-dressed chicken storing system in the cold rooms. Further, most poultry storage was in cold rooms already packaged without direct contact with the storage environment. They made it impossible for cross-contamination from poultry to the cold-room environment or vice versa. The swabbed environment and food can also influence the isolation of the *Listeria* spp. One study revealed a significant difference in isolation of *Listeria* spp. on environmental swabs regarding the type of food processed [59]. This current study has also revealed that carcass contamination does not always mean environmental contamination, such as cold rooms (Table 3).

When the specific part swabbed was considered, a higher prevalence was obtained from exterior swabs indicating higher carcass contamination, possibly because of unhygienic handling practices by food handlers on the processing line. Further, contamination could have arisen from unintended contacts of carcasses with excretions cumulating from the food handlers’ skin, mouth, and nose [60,61]. *Listeria* contamination is suspected to result from cross-contamination of raw processed chicken by improperly cleaned and disinfected processing environments and, to a lesser probability, from the live chickens. The findings of this study concur with similar findings by Cox et al. [62] and Kanarate and others in 2011, who reported processing as a significant hazard of cross-contamination [53]. Comparatively, Ishola and others recorded a higher incidence of 65% on cloacal swabs than on any other parts swabbed [63]. They attributed poultry contaminants to the increased prevalence of contamination with *Listeria*. These findings were like [64,65], stating *Listeria* spp. in soil, water, and hay, including bird guts, as the usual habitat for *L. monocytogenes* [66], including animal feces [67,68].

Clindamycin is widely used in human treatment and animal health to treat Gram-positive bacterial infections [69,70,71]. Antimicrobial resistance in *Listeria* spp. and *L. monocytogenes* differs from when the first report of antibiotic-resistant strains was discovered [66]. In this study, *L*. monocytogenes showed the highest resistance to clindamycin with 61%, and most of these isolates were *L. monocytogenes* accounting for 43% of this total resistance. Followed by tetracycline 30% resistance with all isolates belonging to *L. monocytogenes* and erythromycin (21%) distributed as follows: 13%, 8% *L. monocytogenes* and *L. welshimeri*, respectively. Both abattoirs A and B reported resistant isolates, as seen in Table 5.

Further, from the results of Table 5, there is an indication that resistance was not clustered according to abattoirs but randomly distributed. The results above also suggest the presence of a high level of resistance to clindamycin, tetracycline, and erythromycin, which are antimicrobial agents classified among the most important in veterinary medicine, especially tetracycline [67]. *L. monocytogenes,* from the results in Table 5, remains susceptible to the first drugs of choice in the treatment of Listeriosis as prescribed by CLSI guidelines [32]. However, limited studies have recorded high levels of *L. monocytogenes* resistance to clindamycin, especially from food isolates [68]. Increased clindamycin resistance can influence induction with erythromycin, according to the study by [66]. The mechanism activity of clindamycin is like that of erythromycin, especially in that they share their binding site as recorded by [72]. This clindamycin resistance reported in this current study could partly relate to enzyme inactivation, which is due to possible cross-resistance of the isolation of resistant erythromycin isolates.

This present study also reported erythromycin resistance, contrary to Heelan and others who only reported clindamycin resistance in *Listeria* spp. [72]. The isolation of erythromycin-resistant isolates in this present study could have partly influenced the higher clindamycin resistance recorded due to the possibility of inducible resistance. Although, further analysis needs to be undertaken for a proper conclusion as this present study did not test for resistant genes to help us ascertain this finding.

Tetracycline reveals the second-highest resistance with a 30% prevalence in *L. monocytogenes.* Other authors equally reported similar results to the ones conveyed by this study [73,74]. Equally, a systematic review conducted in R.T.E. foods reported tetracycline as the common antibiotic intrinsically resistant to *L. monocytogenes* classified in the third-generation antibiotics [75]. Furthermore, tetracycline has the most frequently reported resistance phenotype, especially in *L. monocytogenes* sampled from different sources [70,76]. Although, the major factors that might be contributing to enhanced antibiotic resistance could be complex and may point to many existing lapses. The augmented resistance of tetracycline recorded in this study is because of extensive animal feed additives and veterinary treatment [77]. The need to produce more has boosted the increased use of antibiotics such as tetracycline, especially in poultry [78], which is the cheapest source of protein [37]. Inadequate regulation on antibiotic use, particularly in poultry, can partly influence the abuse of certain drugs [78,79]. The other factor links the farm to a folk concept that should ensure the prudent use of antibiotics in animal foods [80].

Similarly, processing procedures are also essential factors that may foster antibiotic resistance in food during farming practices, slaughtering, and transportation [81]. The passing of time has proved that bacteria can develop resistance mechanisms through the evidence presented by other authors in the last decades. Bacteria such as *Listeria* can develop mechanisms or acquired resistance through the transmission of genetic materials coming from other bacterial spp., contributing to the alarming numbers of antimicrobial resistance that may cause threats to the successful treatment of various infectious diseases.

## 4. Materials and Methods

### 4.1. Study Design and Site

We conducted a cross-sectional study design in Lusaka, Zambia, from March 2020 to August 2021. Lusaka has four poultry abattoirs, of which only three enrolled because the other abattoir was closed for renovation. Lusaka has the most significant number of poultry abattoirs because of the recent rapid demand for chicken meat. Additionally, the growing population in the capital city has contributed significantly to the speed of transformation seen in this sector to meet production demand. Moreover, the city attracts several production experts, such as personnel who provide training. The convenience of using antibiotics for the treatment of poultry is among the few things that are helping this industry thrive. The coding of three selected abattoirs was with identification letters as Abattoirs A, B, and C. This cohort sampled only included dressed chickens slaughtered at these abattoirs during the study period.

### 4.2. Sample Size and Sampling

We used a circular systematic random sampling method to select a chicken for swabbing [57]. We collected 150 cloacal and exterior surface swabs, including environmental samples. We estimated the sample size on an assumed prevalence of 27.5% [82] at 80% power and a 5% significance level. The abattoir throughputs ranged from 8000 to 20,000 birds per day. We sampled five dressed chickens per batch each day of sampling. At the three abattoirs, we defined a batch as chickens from the same flock and had homogenous characteristics (i.e., same source, same farm). These sampled abattoirs comprised the target population (N) from the sample population (n). We collected a total of 120 carcass swabs and 30 environmental swabs from the poultry abattoirs. A total of 120 carcasses were randomly selected using the circular systematic random sampling system for each batch by selecting five dressed chickens. We selected 120 carcasses in the three abattoirs at the end of the sampling period for bacteriological examination of *Listeria* and 30 environmental swabs of different batches.

### 4.3. Sample Collection and Processing

We used standard bacteriological sample collection for *Listeria* contamination on samples collected from three abattoirs [83]. The collection of surface swabs was from the cloacal and exterior parts of the carcasses. A sterile metal template outlined a 5 cm^2^ × 5 cm^2^ area of parts marked for swabbing, including environmental swabs. The areas outlined by the metal template denoted the parts for swabbing using a sterile moist cotton gauze wrapped around the end of a flat swab stick. Swabbed samples were in screw-cap tubes containing 6 mL *Listeria* broth [83]. After that, we identified the swabbed samples according to date, ingredient samples (e.g., poultry carcass), batch code, and site name, including comments specific to the sample (e.g., interior or exterior), and were recorded. Identification of samples was by the laboratory code, sampling site/product or ingredient type, date, and site. All samples collected were transported in iceboxes to the microbiology laboratory at the School of the Veterinary Medicine University of Zambia within 3 h of sampling. Upon arrival, we immediately incubated the samples at 37 °C for 48 h. Before isolation from swab diluents, samples were vortexed for 30 s. In environmental and carcass swab samples, they were plated on selective media to detect the target micro-organisms (*Listeria*) [84].

### 4.4. Isolation and Identification of Listeria spp.

We tested poultry carcass swabs for the presence of *Listeria* spp. using the standard international methods recommended by the International Organization for Standardization (ISO g11290-1: 1996, 2004) procedure. First, a 1 g of the sample representative portion from each was inoculated in 9 ml of pre-enriched broth incubated at 37 °C for 24 h, then 1 mL of pre-enriched broth transferred into 9 mL of Fraser broth (Oxoid, Basingstoke, UK) (enriched with *Listeria* selective supplement) and vortexed for 1 min, followed by incubation at 37 °C for 48 h. A loop-full of the Fraser Broth enrichment culture was inoculated on the *Listeria* selective agar (Oxoid) surface, incubated at 37 °C for 48 h, and observed for colonies showing typical greenish sheen growth morphology or green-blue colony color of *Listeria*. The suspected colonies were then sub-cultured onto Nutrient Agar (Oxoid) and later incubated at 37 °C for 24 h to obtain pure colonies. We performed standard biochemical tests on the purified cultures, namely, Gram staining, citrate, urea, indole, motility, oxidase, catalase, and methyl-red tests, to obtain a presumptive diagnosis of *Listeria*.

### 4.5. Phenotypic Detection of Antimicrobial Resistance in Listeria spp. Isolates

The Kirby–Bauer disc diffusion method was used on Mueller Hinton agar with 5% sheep blood on 23 isolates of *Listeria* spp. (Oxoid). Penicillin G (10 unit), gentamicin (30 µg), Levofloxacin (30 µg), erythromycin (15 µg), clindamycin (30 µg), trimethoprim-sulfamethoxazole (1.25/23.75 µg), (30 µg), chloramphenicol (30 µg), and tetracycline (30 µg) were applied as antibiotic agents. CLSI breakpoints for *Listeria* spp. only include a few antimicrobial agents such as trimethoprim-sulfamethoxazole, ampicillin, imipenem, and penicillin; therefore, we used CLSI breakpoints for *Streptococcus* pneumoniae for other antimicrobial agents, and this was because the CLSI guidelines indicated that this was the organism used to interpret breakpoints for *L. monocytogenes* [32]. The identical 23 isolates and antibiotics had their antimicrobial susceptibility tested using the Etest (Biomerieux) with vancomycin and imipenem because there was no interpretation provided under disc diffusion in the CLSI [32]. The quality control strains included were *L. monocytogenes* ATCC 19118, *Streptococcus* pneumoniae ATCC 49619, and *Staphylococcus aureus* ATCC 25923. We classified the isolates as susceptible, intermediate, or resistant in both methods.

### 4.6. PCR Identification of Listeria

The ZYMO DNA extraction kit was used to extract DNA from presumptive *Listeria* spp. colonies. For amplification, a partial fragment of the *prs* gene was targeted on PCR using the primer sequence *prs*-F (5′-GCT GAA GAG ATT GCG AAA GAA G-3′) *prs*-R (5′-CAA AGA AAC CTT GGA TTT GCG G-3′) we used that amplified a fragment of 370 bp [28]. Briefly, One Taq^®^ Quick-Load^®^ from Biolabs and 50 µL reactions of PCR were run. We used a PCR master mix consisting of 25 µL One Taq Quick-load 2X Master Mix with Standard Buffer, 1 uL of 10 µM of each forward and reverse primer, 20 µL of nuclease-free water, and 3 µL of the template. We used thermocycling conditions where initial denaturation was at 94 °C for 1 min, followed by 30 cycles at 94 °C for 45 s, annealing at 53 °C for 45 s, and extension at 72 °C for 2 min, with a final extension step at 72 °C for 5 min. Finally, the expected PCR product band size of 370 bp was visualized on 1.5% agarose gel coated with ethidium bromide.

### 4.7. Purification of PCR Products and Cycle Sequencing

As per the manufacturer’s instructions, we utilized a Promega purification kit to purify the PCR products and a sequencing PCR run using brilliant dye terminator V3.1 (Wizard S.V. Gel & PCR Promega clean-up System). The sequencing products were further precipitated using the ethanol precipitation method, followed by denaturation and loading in ABI 3500 Genetic analyzer for sequencing [85].

### 4.8. Sequence Analysis

We first assembled and edited the sequences obtained from this study using the ATGC plug-in in Genetyx ver. 12 genetyx corporation, Tokyo, Japan. The assembled sequences were then subjected to BLAST analysis on the NCBI website (https://blast.ncbi.nlm.nih.gov/Blast.cgi (accessed on 12 February 2022)) to identify the spp. of bacteria isolated and sequenced. All sequences generated in this study were deposited in Japan’s DNA Database with accession numbers LC700404 to LC700426.

## 5. Conclusions

This study aimed to determine the prevalence of *Listeria* spp. isolated from poultry abattoirs in Lusaka and determine the resistant patterns of the isolated *Listeria* spp. The results indicate that *L. monocytogenes* was the most common isolated spp. Further findings revealed, phenotypically, that clindamycin, tetracycline, and erythromycin had the highest prevalence of antibiotic resistance through the first drugs of choice and remained susceptible. These results are essential for determining poultry safety and treatment of Listerioses by antibiotics. The results can contribute significantly to the prudent use of antibiotics in poultry and possibly prevention of future acquisition of resistance by *L. monocytogenes.* Furthermore, this study is the first to have isolated *Listeria* spp. in poultry carcasses using PCR to amplify the *prs* gene to confirm the presence of *Listeria* spp. in Zambia. Equally, there is a need to strengthen the observed phenotypic antibiotic resistance recorded in this current study by screening for resistant genes using PCR methods.

## Figures and Tables

**Table 1:** Prevalence of *Listeria* spp. in dressed poultry and environmental samples (*n* = 150).

Variable	*n*	Frequency	Prevalence %	95% CI	*p*-Value
Environmental swabs	30	3	2	0.52–6.19	<0.001
Carcass swabs	120	20	13	8.53–20.08	<0.001
Overall	150	23	15	10.16–22.33	<0.001

*n* = number of swabs; 95% CI = Confidence Interval; *p*-value = 0.05.

**Table 2:** Description and BLAST analysis of samples collected from poultry abattoirs in Lusaka.

Site of Swabbing	Abattoir	*Listeria* spp.	Accession Number	Blast Analysis Similarity Score (%)	Reference Accession Number
Cloacal	A	*L. welshimeri*	LC700404	97	LT906444.1
Cloacal	A	*L. monocytogenes*	LC700405	94	MK883765.1
Cloacal	A	*L. monocytogenes*	LC700406	99.39	MK883765.1
Cloacal	A	*L. monocytogenes*	LC700407	99.39	MK883765.1
Cloacal	A	*L. monocytogenes*	LC700408	99.38	MK883765.1
Exterior surface	A	*L. monocytogenes*	LC700410	99.39	MK883765.1
Exterior surface	A	*L. monocytogenes*	LC700412	99.70	MK883765.1
Exterior surface	A	*L. monocytogenes*	LC700413	98.79	MK883765.1
Exterior surface	A	*L. monocytogenes*	LC700414	100.00	MK883765.1
Exterior surface	A	*L. monocytogenes*	LC700415	98.79	MK883765.1
Exterior surface	A	*L. welshimeri*	LC700411	99.39	CP065605.1
Exterior surface	A	*L. monocytogenes*	LC700416	99.02	CP054846.1
Exterior surface	A	*L. monocytogenes*	LC700418	98.79	MK883765.1
Exterior surface	A	*L. monocytogenes*	LC700420	98.48	CPO54846.1
Exterior surface	A	*L. monocytogenes*	LC700421	99.37	MK883765.1
Exterior surface	A	*L. monocytogenes*	LC700422	100.00	AY1135439.1
Exterior surface	A	*L. monocytogenes*	LC700423	94.24	MK883763.1
Environmental swab	B	*L. innocua*	LC700409	99.70	KC808562.1
Cloacal	B	*L. welshimeri*	LC700417	98.18	CP065605.1
Exterior surface	B	*L. welshimeri*	LC700419	96.54	CPO65605.1
Environmental swab	B	*L. welshimeri*	LC700424	99.39	KC808562
Environmental swab	B	*L. monocytogenes*	LC700425	99.39	CPO65605.1
Exterior surface	B	*L. monocytogenes*	LC700426	98.79	CP065605.1

**Table 3:** Distribution of specific *Listeria* species across different abattoirs from the carcass and environmental swabs (*n* = 23).

Abattoir Code	*Listeria* spp. (23)	Environmental Swabs	Carcass Swabs	Prevalence %
**A**	*L. monocytogenes*	-	15	65
	*L. innocua*	-	-	-
	*L. welshimeri*	-	2	9
**Totals**		-	17	73
**B**	*L. monocytogenes*	1	1	8
	*L. innocua*	1	-	4
	*L. welshimeri*	-	3	13
**Totals**		2	4	26
**C**	*L. monocytogenes*	-	-	-
	*L. innocua*	-	-	-
	*L. welshimeri*	-	-	-
**Totals**		-	-	-

**Table 4:** Isolation of *Listeria* spp. from the different surfaces swabbed (*n* = 23).

*Listeria* spp.	Surfaces Swabbed		
	Cloacal %	Exterior Surface %	Environmental Swabs %
*L. monocytogenes*	4	12	1
*L. innocua*	-	-	1
*L. welshimeri*	2	2	1
Totals	6 (26)	14 (61)	3 (13)

**Table 5:** Antimicrobial resistance of *Listeria* species isolated from raw dressed broilers (*n* = 23).

	(Disc Diffusion) *n* = 23			(Etest) *n* = 23		
Antibiotics	R (%)	I (%)	S (%)	R (%)	I (%)	S (%)
Penicillin G	8 (35)	14 (61)	1 (4)	1 (4)	2 (9)	20 (87)
Imipenem	-	-	-	0.0	14 (61)	9 (39)
Gentamicin	2 (9)	2 (9)	19 (82)	0	0	100
Levofloxacin	3 (13)	-	20 (87)	0	0	100
Trimethoprim/sulfamethoxazole	8 (33)	0	15 (65)	0.0	1 (4)	22 (96)
Clindamycin	13 (57)	2 (9)	8 (35)	14 (61)	0	9 (39)
Erythromycin	5 (22)	2 (9)	16 (70)	5 (22)	10 (42)	9 (38)
Vancomycin	-	-	-	0	0	100
Chloramphenicol	3 (13)	0	20 (87)	0	0	100
Tetracycline	13 (57)	3 (13)	7 (30)	7 (30)	1(4)	15 (65)

(R) means resistance, (I) means intermediate, and (S) means susceptible.

## Data Availability

All the sequences from this study were deposited in the DNA Database of Japan with accession numbers LC700404 to LC700426. All the data regarding this manuscript we contained within the text.
